# Antiepileptic Effect of *Uncaria rhynchophylla* and *Rhynchophylline* Involved in the Initiation of c-Jun N-Terminal Kinase Phosphorylation of MAPK Signal Pathways in Acute Seizures of Kainic Acid-Treated Rats

**DOI:** 10.1155/2013/961289

**Published:** 2013-12-04

**Authors:** Hsin-Cheng Hsu, Nou-Ying Tang, Chung-Hsiang Liu, Ching-Liang Hsieh

**Affiliations:** ^1^Graduate Institute of Chinese Medicine, College of Chinese Medicine, China Medical University, Taichung 40402, Taiwan; ^2^Department of Chinese Medicine, China Medical University Hospital, Taichung 40402, Taiwan; ^3^Department of Neurology, China Medical University Hospital, Taichung 40402, Taiwan; ^4^Acupuncture Research Center, China Medical University, Taichung 40402, Taiwan; ^5^Graduate Institute of Integrated Medicine, College of Chinese Medicine, China Medical University, 91 Hsueh-Shih Road, Taichung 40402, Taiwan

## Abstract

Seizures cause inflammation of the central nervous system. The extent of the inflammation is related to the severity and recurrence of the seizures. Cell surface receptors are stimulated by stimulators such as kainic acid (KA), which causes intracellular mitogen-activated protein kinase (MAPK) signal pathway transmission to coordinate a response. It is known that *Uncaria rhynchophylla* (UR) and *rhynchophylline* (RP) have anticonvulsive effects, although the mechanisms remain unclear. Therefore, the purpose of this study is to develop a novel strategy for treating epilepsy by investigating how UR and RP initiate their anticonvulsive mechanisms. Sprague-Dawley rats were administered KA (12 mg/kg, i.p.) to induce seizure before being sacrificed. The brain was removed 3 h after KA administration. The results indicate that pretreatment with UR (1.0 g/kg), RP (0.25 mg/kg), and valproic acid (VA, 250 mg/kg) for 3 d could reduce epileptic seizures and could also reduce the expression of c-Jun aminoterminal kinase phosphorylation (JNKp) of MAPK signal pathways in the cerebral cortex and hippocampus brain tissues. Proinflammatory cytokines interleukin (IL)-1**β**, IL-6, and tumor necrosis factor-**α** remain unchanged, indicating that the anticonvulsive effect of UR and RP is initially involved in the JNKp MAPK signal pathway during the KA-induced acute seizure period.

## 1. Introduction

Inflammatory processes play a critical role in neurodegenerative disorders such as stroke, epilepsy, and Alzheimer's disease [1]. Seizure can cause inflammation of the central nervous system, which can affect the severity and frequency of recurring seizures [2]; moreover, anti-inflammatory effects such as those caused by a ketogenic diet, can produce anticonvulsant effects [2]. Previous research has reported that seizure induces the generation of cytokines, such as interleukin (IL)-1*β*, IL-6, and tumor necrosis factor (TNF)-*α*, and that the interactions among these cytokines are related to the development of epilepsy and play a crucial role in epileptogenesis [3]. Nuclear factor (NF)-*κ*B is a transcription factor that can be activated by TNF-*α* and the Fas ligand. NF-*κ*B presents in the cytosol in an inactive form as a 3-subunit complex comprising p65/p50 dimers and I*κ*B [4]. NF-*κ*B is activated by phosphorylation of I*κ*B*α* and subsequently translocates into the nucleus where it binds to form NF-*κ*B-DNA [4]. Long-lasting seizures can also cause NF-*κ*B expression and translocation [5]. Furthermore, the activation of NF-*κ*B can contribute to susceptibility to seizures [6]. The glial NF-*κ*B activation can induce the generation of proinflammatory cytokines and neurotoxic reactive oxygen species, resulting in neuronal apoptosis [4]. Therefore, NF-*κ*B plays multiple roles in inflammation.

Cells can transmit intracellular signals to coordinate a response to external stimuli [7]. Cell surface receptors such as the N-methyl-D-asparate (NMDA) and kainate (non-NMDA) receptors of glutamate play a crucial role in epilepsy because they can cause activation of mitogen-activated protein kinase (MAPK), which plays a critical role in the development of epilepsy [8]. The MAPK signal pathways comprise the following 3 main subfamilies that are activated by phosphorylation [7]: (1) extracellular signal-regulated protein kinase (ERK); (2) c-Jun aminoterminal kinase (JNK)/stress-activated protein kinase (SAPK); and (3) p38.

*Uncaria rhynchophylla* (UR) is a traditional Chinese herb that has been used to treat epileptic seizures for centuries. According to the recording of Bencoogongmu, the UR can treat vertigo and epileptic seizure [9]. Our previous study showed that UR can reduce epileptic seizures [10]. It can also reduce microglial activation, neuronal and inducible nitric oxide synthase immunoreactive cells of the hippocampus in kainic acid (KA)-treated rats, and also acts as a neuroprotector [11]. In addition, our previous research indicated that UR and its *rhynchophylline* (RP) component can attenuate JNKp expression and NF-*κ*B activation at 24 hr following KA-induced epileptic seizures [12]. However, following our previous studies [12], further clarification is required to identify the initiating event during the seizures and whether this initiator was possibly related to the development of epilepsy (epileptogenesis). Therefore, to develop a novel strategy for treating epilepsy, the purpose of this study is to investigate the anticonvulsive mechanisms of UR and its RP component during acute seizures. In this study, Sprague-Dawley (SD) rats were pretreated with UR and RP. Because the findings of our previous studies [10, 12] showed that peritoneal administration of KA can cause acute seizures at 3 h, in this study the concentrations of the proinflammatory cytokines IL-1*β*, IL-6, and TNF-*α*, as well as the MAPKs and NF-*κ*B-activation of brain tissues, were measured at 3 h following KA administration.

## 2. Material and Methods

### 2.1. Preparation of UR Extract

Crude UR [*Rubiaceae, Uncaria rhyncholhylla* (Miq.) jack] was extracted by following our previous study [12]. The crude UR was extracted twice (500 g each) with 3.5 and 2.5 L of distilled water by boiling for 1 h. The extract was filtered twice and freeze-dried, and the total yield was 63.55 g (12.71%). The freeze-dried extract was stored in a refrigerator at 4°C and was analyzed using a high-performance liquid chromatography system (Hitachi Instruments Service Co. Ltd., Interface D-700, Pump L-7100, UV-Vis Dector L-7420, Ibarki-Ken, Japan) with RP (Matsumura Yakugyo Co., Ltd, Japan) as a standard from Koda Pharmaceutical Company. Because 1 g of the freeze-dried UR extract contained 0.22 mg of RP, the dosage of RP employed in this study was 0.25 mg/kg.

### 2.2. Animal Models

The adult male SD rats (200–300 g) employed in this study were purchased from Biol-Asco Taiwan Co. Ltd. The rats were housed in the animal center of China Medical University, under a 12-h light-dark cycle (25°C ± 1°C), and provided with food and water. All of the procedures in the experiments were conducted in accordance with the *Guideline Principles for the Care and Use of Laboratory Animals.*

### 2.3. Electrode Preparation

The electrodes were prepared 1 wk prior to electroencephalogram (EEG) and electromyogram (EMG) recordings, in accordance with our previous research [12]. The head of the rats was fixed in a stereotactic apparatus under an anesthetic state (chloral hydrate 400 mg/kg, i.p.). The hair of the rat head was cut using scissors. Using a surgical knife, an incision was made on the scalp from the midline and the skull was exposed. The stainless screw electrodes were implanted in the bilateral sensorimotor cortices just as locate epidural service as the recordings, and another electrode was located in the frontal sinus as a reference for the EEG recordings. For the EMG recordings, bipolar electrode wires were placed around the neck muscle through the subcutaneous tissues. Finally, all of the electrodes were plugged into a relay and then connected to an EEG and EMG recording machine (MP100WSW, BIOPAC System, Inc. Goleta, CA, USA).

### 2.4. Experimental Procedure

The 36 SD rats examined in this study were randomly divided into 6 groups (6 rats/group), detailed as follows: (1) normal group (Group N), phosphate-buffered saline (PBS) solution 1.0 mL/kg i.p only; (2) control group (Group KA), KA (12 mg/kg; King Don Co., Taiwan) i.p. only; (3) PK (Group PK), PBS (1.0 mL/kg/day, i.p.) for 2 d, as well as 15 min prior to KA administration; (4) UR (Group UR), UR (1.0 g/kg/day, i.p.) for 2 d, as well as 15 min prior to KA administration; (5) RP (Group RP), RP (0.25 mg/kg/d, i.p.) for 2 d, as well as 15 min prior to KA administration; and (6) VA (Group VA), VA (250 mg/kg/d, i.p.; Sigma USA) for 2 d, as well as 15 min prior to KA administration. The epileptic seizures were confirmed by behavioral observation and EEG and EMG recordings. The EEG and EMG were recorded 15 min prior to drug administration until 3 h following KA administration, and the rats were sacrificed under anesthesia (chloral hydrate, 400 mg/kg, i.p.). The rat brain was removed and separated into the cortex and hippocampus regions to measure the IL-1*β*, IL-6, and TNF-*α*, as well as the JNK, ERK, and p38 of MAPK and NF-*κ*B.

### 2.5. Rat Brain Tissue Preparation

The brain tissue was washed twice under cold PBS solution and then dried. Subsequently, 200 *μ*L 0.25 × buffer H (HEPES-KOH pH 7.9, 100 mM; KCl, 250 mM; Spermine, 1.5 mM; Spermine, 5 mM; EGTA, 10 mM; EDTA, 10 mM), 0.5 mM DTT, 1 × protease inhibitor mixture (PMSE, 50 mM; Benzamidine, 0.1 M; leupeptin, 50 *μ*g/mL; pepstatin, 100 *μ*g/mL), and 1 × phosphatase inhibitor mixture (okadaic acid, 200 nM; sodium orthovanadate, 20 mM; cypermethrin, 8 nM) were added. The sample was transferred to a new centrifuge tube, and 2 × buffer H, 20% glycerol, 0.5 mM DTT, 1 × protein inhibitor mixture, and 1 × phosphatase inhibitor were added, the volume of which was identical to that of the brain tissue. The sample mixture was homogenised and centrifuged (2500 rpm) at 4°C for 10 min to dislodge the supernatants. The sample was added into an identical volume of low-salt buffer (1 × buffer H, 20% glycerol, 1 mM DTT, 1 × protease inhibitor mixture, 1 × phosphate inhibitor mixture) and subsequently added to a high-salt buffer (1 × buffer H, 1 M KCl, 20% glycerol, 1 mM DTT, 1 × protease inhibitor mixture, 1 × phosphate inhibitor mixture) that was equal to 0.72 × of the sample. Next, the sample was extracted at 4°C for 45 min. Finally, the sample was centrifuged (12 000 rpm) at 4°C for 5 min, and the supernatants were stored in a refrigerator at −70°C. The concentration of protein was determined using a Bradford assay.

### 2.6. Enzyme-Linked Immunosorbent Assay (ELISA)

The IL-1*β* (Bender MedSystems, USA), IL-6 (Bender MedSystems, USA), and TNF-*α* (Bender MedSystems, USA) kits were added to various concentrations of supernatants for the protein marker. Subsequently, a colorimetric method (optical density) was employed using the ELISA reader (Dynex MRX, Virginia, USA) to determine the concentration of IL-1*β*, IL-6, and TNF-*α*.

### 2.7. Western Blotting Analysis

Western blotting analysis was performed by following our previous study [12]. Briefly, the brain tissue was homogenized in lysis buffer solution (pH 7.2 Tris-HCl, 50 mM; 5% Triton X-100; 1 × protease inhibitors). The homogenous brain tissue was centrifuged (12 000 rpm) at 4°C for 4 min, and the supernatants were then stored in a refrigerator at −70°C until the AP-1 activity was determined. The protein extracts (10 *μ*g) were separated using 10% sodium dodecyl sulfate-polyacrylamide gel electrophoresis (SDS-PAGE), and the protein bands were subsequently transferred electrophoretically to nitrocellulose membranes. The membranes were blocked in blocking buffer solution (pH 7.6 Tris-HCl, 20 mM; NaCl, 140 mM; Tween-20, 0.1%; skim milk powder, 5%) and probed with antibodies that recognized phosphorylated ERK (ERKp), JNKp, phosphorylated p38 (p38p), ERK, JNK, or p38. The bound antibody was detected using peroxidase-conjugated anti-rabbit antibody followed by chemiluminescence (ECL system, Amersham, Buckinghamshire, UK) and exposure to film. We used the software (AlphaEase FC) to calculate the density of protein expression from Western blot assay and then JNK as a denominator and JNKp as numerator. Similarly, ERK and p38 are the denominator, and ERKp and p38p are numerator to quantitate the protein express.

### 2.8. Electrophoretic Mobility Shift Assay

Electrophoretic mobility shift assay (EMSA) was conducted in accordance with our previous study [12]. The nuclear extract (10 *μ*g for protein) was incubated in a binding buffer solution with double-stranded biotin-labeled oligonucleotide probes at 25°C for 30 min and then separated using 6% polyacrylamide gel (40% bis/acryl amide (19 : 1), 1.5 mL; 10 × TBE, 250 *μ*L; 10% APS, 50 *μ*L; TEMED, 5 *μ*L; DDH_2_O, 8.25 mL) electrophoresis (30 V) for 2.5–3.0 h. The protein bands were then transferred to nylon membranes for 1 h, which were irradiated in a UV cross-linking apparatus. Subsequently, the membranes were blocked using a blocking buffer solution (10 × maleic acid buffer, 13 mL; 10 × blocking solution, 13 mL; DDH_2_O, 100 mL) and probed using alkaline phosphate-conjugated streptavidin. The membrane was washed twice with washing buffer solutions. The oligonucleotides were detected using CSPD and exposure to film.

### 2.9. Statistical Analysis

The data are represented as mean (± standard deviation, SD). The groups were compared by oneway analysis of variance (ANOVA), followed by Tukey's test. Any *P* values < .05 were considered statistically significant.

## 3. Results

### 3.1. Effect of UR, RP, and VA on the Animal Models of KA-Induced Epileptic Seizures in SD Rats

**Figure 1:**
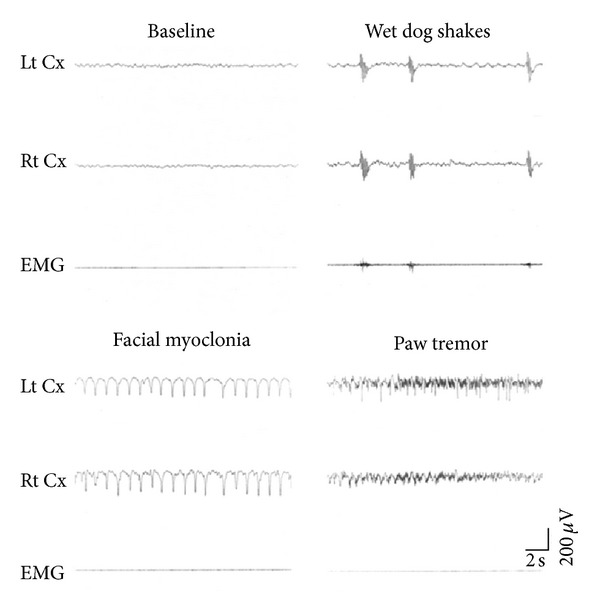
KA-induced epileptic seizure and EEG and EMG changes. KA (i.p.) in rats caused 4 types of epileptic seizures: wet dog shakes with polyspike-like artifacts on the EEG and EMG, facial myoclonia with continuous sharp waves on the EEG, and paw tremor with continuous spikes on the EEG. Lt Cx: EEG recorded at left cerebral cortex. Rt Cx: EEG recorded at right cerebral cortex. Baseline: EEG and EMG recordings prior to KA administration.

Peritoneal administration of KA in the SD rats caused epileptic seizure, and their main behaviors included wet dog shakes (WDS) with polyspike-like artifacts observed on the EEG and EMG, facial myoclonia (FM) with continuous sharp waves on the EEG, and paw tremor (PT) with continuous EEG spikes (Figure 1).

**Figure 2:**
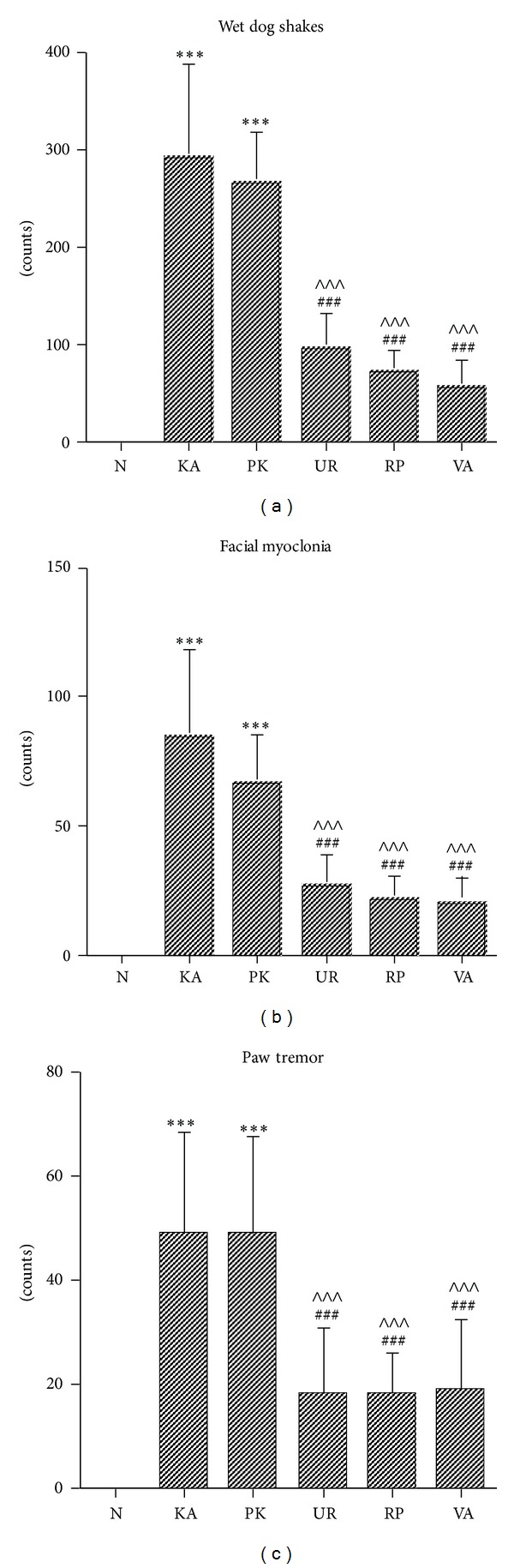
Effect of UR and RP on animal models of KA-induced epileptic seizures in rats. KA (i.p.) increased the frequency of wet dog shakes, facial myoclonias, and paw tremors; these increases were lower in Groups UR, RP, and VA (N, normal group; KA, KA/control group; PK, PK group; UR, UR group; RP, RP group; VA, VA group). ***P < .001 compared with N; ^###^ *P* < .001 compared with KA; ^∧∧∧^ *P* < .001 compared with PK.

The WDS counts for Groups KA and PK increased significantly compared with those for Group N (both *P* < .001; Figure 2). These increases were lower in Groups UR, RP, and VA (all *P* < .001; Figure 2). Similar WDS counts were observed between Groups KA and PK, UR and RP, and RP and VA (all *P* > .05; Figure 2).

The FM counts for Groups KA and PK increased significantly compared with those for Group N (both *P* < .001; Figure 2). These increases were lower in Groups UR, RP, and VA (all *P* < .001; Figure 2). The FM counts were similar between Groups KA and PK, UR and RP, and RP and VA (all *P* > .05; Figure 2).

The PT counts for Groups KA and PK were significantly higher than those for Group N (both *P* < .001; Figure 2). These increases were lower in Groups UR, RP, and VA (all *P* < .001; Figure 2). The PT counts for Groups KA and PK were similar, as well as UR and RP, and RP and VA (all *P* > .05; Figure 2).

### 3.2. Effect of UR, RP, and VA on IL-1*β*, IL-6, and TNF-*α* in the Animal Models of KA-Induced Epileptic Seizures in SD Rats

**Figure 3:**
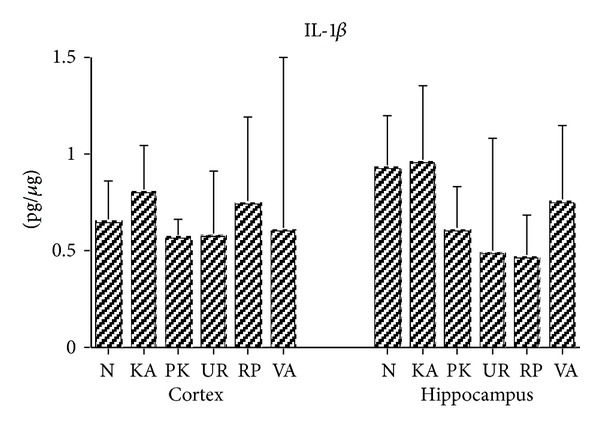
Effect of UR and RP on IL-1*β* in KA-induced epileptic seizures in rats. The IL-1*β* levels in the cerebral cortex (Cortex) and hippocampus (Hippocampus) brain tissues were similar in Groups N, KA, PK, UR, RP, and VA (N: normal group; KA: KA/control group; PK: PK group; UR: UR group; RP: RP group; VA: VA group).

The IL-1*β* levels in the cerebral cortex and hippocampus brain tissues were similar between two groups in Groups N, KA, PK, UR, RP, and VA at 3 h following KA administration (all *P* > .05; Figure 3).

**Figure 4:**
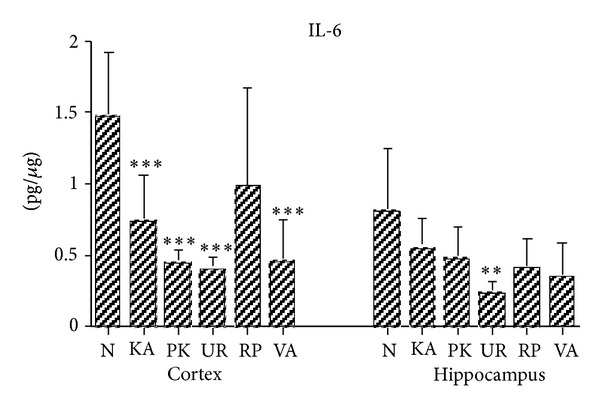
Effect of UR and RP on IL-6 in KA-induced epileptic seizures in rats. The IL-6 levels were lower in the cerebral cortex (Cortex) brain tissue for Groups KA, PK, UR, and VA than those in Group N. The IL-6 levels in the hippocampus (Hippocampus) brain tissue for Group UR were lower than those in Group N (N, normal group; KA, KA/control group; PK, PK group; UR, UR group; RP, RP group; VA, VA group). JNK: *P < .05, ***P < .001 compared with N.

The IL-6 levels in Group N were significantly higher than those in Groups KA, PK, UR, and VA (all *P* < .001; Figure 4). However, the IL-6 levels for Groups N and RP were similar (*P* > .05; Figure 4). The IL-6 levels in the cerebral cortex brain tissues were similar between two groups in Groups KA, PK, UR, RP, and VA at 3 h following KA administration (all *P* > .05; Figure 4).

The IL-6 levels in Group N were significantly higher than those in Group UR (*P* < .05; Figure 4). The IL-6 levels in the hippocampus of brain tissue were similar between two groups in Groups N, KA, PK, RP, and VA at 3 h after KA administration (all *P* > .05; Figure 4).

**Figure 5:**
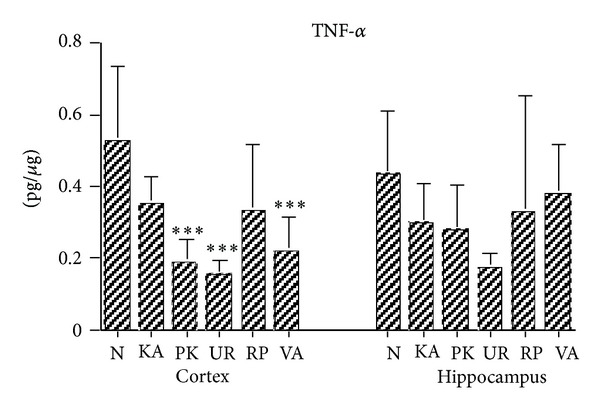
Effect of UR and RP on TNF-*α* in KA-induced epileptic seizures in rats. The TNF-*α* level in the cerebral cortex (Cortex) brain tissue for Groups PK, UR, and VA were lower than those in Group N. The TNF-*α* levels in the hippocampus (Hippocampus) brain tissue were similar in Groups N, KA, PK, UR, RP, and VA (N: normal group; KA: KA/control group; PK: PK group; UR: UR group; RP: RP group; VA: VA group). ***P < .001 compared with N.

The TNF-*α* levels in Group N were significantly higher than those in Groups PK, UR, and VA (all *P* < .001; Figure 5). The TNF-*α* levels in Groups N, KA, and RP were similar (all *P* > .05; Figure 5). The TNF-*α* levels in the cerebral cortex brain tissues were similar between two groups in Groups PK, UR, and VA at 3 h after KA administration (all *P* > .05; Figure 5).

The TNF-*α* levels in the hippocampus brain tissues were similar between two groups in Groups N, KA, PK, UR, RP, and VA at 3 h following KA administration (all *P* > .05; Figure 5).

### 3.3. Effect of UR, RP, and VA on JNK, ERK, p38 MAPK, and NF-*κ*B in the Animal Models of KA-Induced Epileptic Seizures in SD Rats

**Figure 6:**
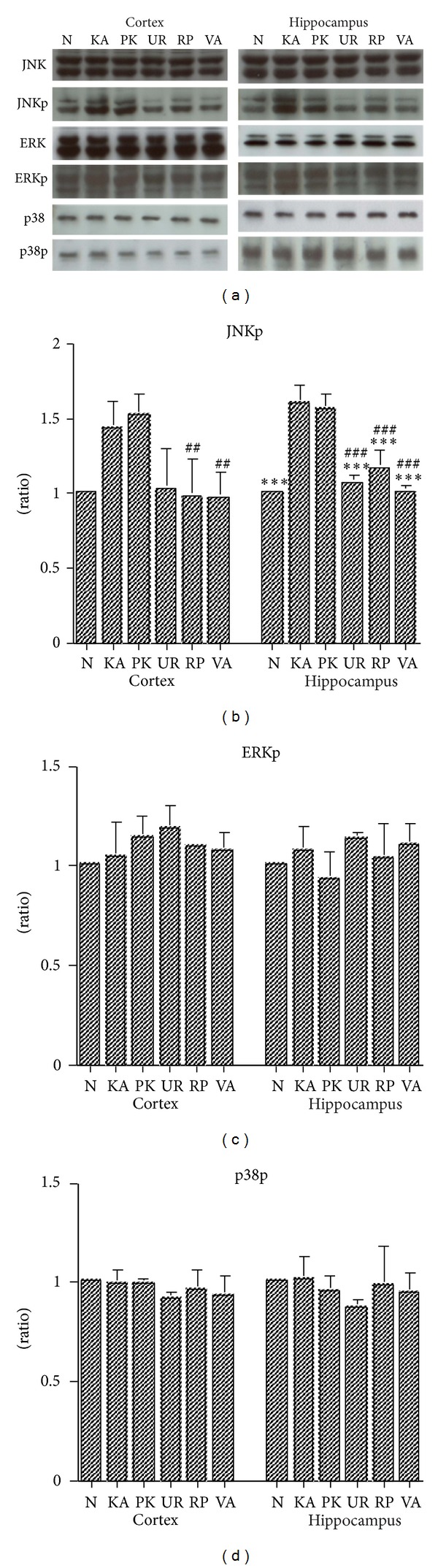
Effect of UR and RP on MAPK in KA-induced epileptic seizures in rats. The JNKp expression of MAPK in the cerebral cortex (Cortex) and hippocampus (Hippocampus) brain tissues increased for Groups KA and PK; these increases were lower in Groups UR, RP and VA. The JNK, ERK, ERKp, p38, p38p expression of MAPk in the cerebral cortex and hippocampus brain tissues were similar in Groups N, KA, PK, UR, RP, and VA (p: phosphorylation; N: normal group; KA, KA/control group; PK: PK group; UR: UR group; RP: RP group; VA: VA group). ^##^ *P* < .01, ^###^ *P* < .001 compared to PK; ***P < .001 compared to KA.

In the cerebral cortex brain tissues, an increase in the JNKp expression of MAPK signal pathways was observed in Groups KA and PK, whereas those increases were lower in Groups UR, RP, and VA at 3 h following KA administration (Figure 6).

In the hippocampus brain tissues, an increase in the JNKp expression of MAPK signal pathways was observed in Groups KA and PK, whereas these increases were lower in Groups UR, RP, and VA at 3 h following KA administration (Figure 6).

In both the cerebral cortex and hippocampus brain tissues, the ERKp and p38p expressions of MAPK signal pathways were similar in Groups N, KA, PK, UR, RP, and VA at 3 h following KA administration (Figure 6).

**Figure 7:**
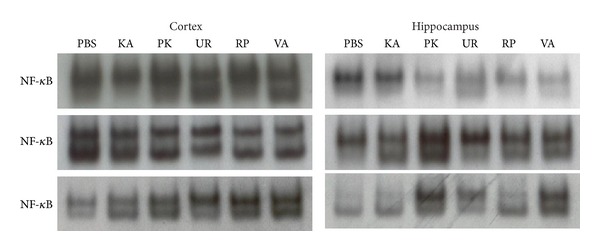
Effect of UR and RP on NF-*κ*B activity in KA-induced epileptic seizures in rats. The NF-*κ*B activity was no conclusive because of inconsistent results following 3 repeated experiments from EMSA. N: normal group; KA: KA/control group; PK: PK group; UR: UR group; RP: RP group; VA: VA group.

Regarding the NF-*κ*B activity, no conclusive evidence was obtained because of the inconsistent results following 3 repeated experiments (Figure 7).

## 4. Discussion

The results showed that pretreatment using UR, RP, and VA reduced the WDS, FM, and PW counts during the epileptic seizures; indicating that UR, RP, and VA have anticonvulsive effects on animal models of KA-induced epileptic seizures in SD rats; furthermore, these results are in good agreement with those from our previous study [12]. Our previous proteomic study showed that under expression of macrophage migration inhibitory factor (MIF) and cylcophilin proteins in either the cerebral cortex or hippocampus brain tissues of KA-treated rats, whereas those under expression could be reversed by administering UR or RP as a pretreatment [13]. Treatment with UR can reduce population spikes in hippocampal neuronal cells, and it can also reduce glial proliferation and S100 protein expression [14]; in addition, it can attenuate mossy fiber sprouting in animal models of KA-induced epileptic seizures in rats [15]. These findings indicate that pretreatment using UR, RP, and VA can suppress the development of epilepsy.

Brain damage such as that caused by encephalitis induces inflammatory responses that can promote neuronal excitability, which contributes to the generation of seizures. Consequently, the inflammation becomes exacerbated and results in epileptogenesis [2]. IL-1*β* is produced in injured brain tissue, and the bonding of IL-1*β* to the IL-1 receptor can initiate the MAPK signaling pathways and NF-*κ*B activation [16]. Our results show that the IL-1*β* levels in the cerebral cortex and hippocampus brain tissues were similar between two groups in Groups N, KA, PK, UR, RP, and VA at 3 h after KA administration. Pernot et al. [17] reported that the levels of IL-1*β* mRNA increased between 5 and 24 h following KA intrahippocampal injection.

The results of this study show that the IL-6 levels were greater in Group N than those in Groups KA, PK, UR, and VA, whereas the IL-6 levels in the cerebral cortex brain tissues were similar between two groups in Groups KA, PK, UR, RP, and VA; however, the IL-6 levels in Group N were greater than those in Group UR, whereas the IL-6 levels in the hippocampus brain tissues were similar between two groups in Groups N, KA, PK, RP, and VA at 3 h following KA administration. Based on these results, we considered whether the IL-6 levels in the cerebral cortex and hippocampus brain tissues were unchanged at 3 h following KA administration. Uludag et al. [18] showed that the serum levels of IL-6 and IL-1 receptor antagonist (IL-1Ra) increased at 12 h following seizures in 23 epilepsy patients. Lehtimäki et al. [19] reported that the increased IL-6 levels in the cerebrospinal fluid (CSF) and serum following seizures were greater in recurrent generalized tonic-clonic seizure (GTS) patients than those in either single GTS or prolonged partial seizure patients; therefore, they concluded that seizures could induce cytokine production and that the CSF and serum IL-6 levels might correlate with the severity of a seizure. A recurrent seizure can induce IL-6 production, which can cause changes in neuronal tissue, resulting in the development of refractory seizures.

The results of this study indicated that the TNF-*α* levels in the cerebral cortex brain tissues for Group N were greater than those in Groups PK, UR, and VA, although the TNF-*α* levels were similar between two groups in Groups N, KA, and RP, as well as between two groups in Groups PK, UR, and VA; however, the TNF-*α* levels in the hippocampus brain tissues were similar between two groups in Groups N, KA, PK, UR, RP, and VA at 3 h following KA administration. Accordingly, we considered whether the TNF-*α* levels in the cerebral cortex and hippocampus brain tissues had not changed at 3 h following KA-induced epileptic seizures. Previous research reported that the TNF-*α* mRNA peaked at 6 h following limbic seizure with epilepticus [20]. TNF-*α* plays dual roles of anti- and proconvulsion, which is mediated by its neuronal p75 receptor, which can inhibit seizures and exert an anticonvulsive effect. In contrast, another study reported that TNF-*α* was mediated by the p55 receptor, which acted as a proconvulsant [21]. This discussion shows that the levels of IL-1*β*, IL-6, and TNF-*α* did not increase at 3 h following KA administration in this study, indicating that timing is a critical factor in the production of IL-1*β*, IL-6, and TNF-*α*. Our results indicated that UR, RP, and VA could reduce epileptic seizures, although they did not change the levels of proinflammatory cytokines IL-1*β*, IL-6, and TNF-*α*. Therefore, we infer that the anticonvulsive effect of UR, RP, and VA—at least for proinflammatory cytokines IL-1*β*, IL-6, and TNF-*α*—did not play a key role in the KA-induced acute seizure.

Our results also indicated that the JNKp expression of the MAPK signal pathway increased in the cerebral cortex and hippocampus in Groups KA and PK. These increases can be attenuated by pretreatment using UR, RP, and VA for 3 d. Previous research showed that MAPKs play a critical role in cell differentiation, proliferation, and death and is activated by phosphorylation cascades [22]. KA is an analogue of glutamate, and can induce limbic seizures. Furthermore, previous research has shown that KA can induce the activation of JNK and p38, which are both involved in cell death [23]. The MAPK protein and its active form increase between 30 and 60 min following pilocarpine-induced epilepticus. Previous research hypothesized that this activation could contribute to the mechanisms of acute epileptogenesis and long-lasting changes of neuropathology [24]. Che et al. [25] reported that p38 MAPK of brain tissue increased at 4 d following KA-induced seizures in mice and that this p38 MAPK signal pathway plays a crucial role in either neuronal death or reactive gliosis. Previous studies have shown that ERK MAPK plays a critical role in cell death [26] and that the MAPK signal pathway plays a critical role in the physiological and biochemical regulation of NMDA receptors [27]. Sokka et al. [28] reported that KA activates non-NMDA receptor and that induced seizures can cause stress of the endoplasmic reticulum (ER), resulting in the accumulation of unfolded protein. Furthermore, this stress of the ER has been shown to cause the activation of JNK, which is similar to cell surface receptor reactions to extracellular signal stimulation [29]. KA can induce the release of glutamate from neuronal cells and can also induce the ER stress of astrocyte. Chihara et al. [30] reported that the old astrocyte specifically induced substance (OASIS) in astrocyte can respond to the ER stress and that it played a protective role in KA-induced ER stress. Lee et al. [31] showed that pretreatment using an alkaloid fraction of UR can block NMDA-induced cytotoxicity, which acts as a neuroprotector by inhibiting apoptosis. In addition, in this study, the results for the NF-*κ*B levels were inconclusive because of the inconsistent results following the 3 experiments; the levels of NF-*κ*B were the upstream of the pro-inflammatory cytokines IL-1*β*, IL-6, and TNF-*α*. Previous studies have reported that the DNA-binding activity of NF-*κ*B increased at 24 h following KA-treatment in rat models [10, 32]. In summary, we assert that the JNKp MAPK signal pathway plays a critical role in epileptogenesis at 3 h following KA administration; therefore, UR, RP, and VA suppression of the expression of JNKp MAPk inhibit the development of epilepsy. Thus, we suggest that the present study is more advantage to develop the novel antiepileptic drug than our previous study [12]. Because the results indicated that JNKp MAPK signal pathway involve to acute seizure.

Some limitation in the present study is as follows: (1) the dose of UR and RP is difficult to determine due to high dose possible reduce the effect of UR; (2) although UR is most commonly used for the treatment of vertigo and epilepsy in Taiwan and in our clinic and according to our knowledge, the severe side effect and herb-drug interaction were not found. The further observation and study still remain to be needed; (3) the present study limits in animal model level. Therefore, how to design a randomized, double blind, placebo-controlled clinical trial in epileptic patient is an import issue in the future.

In conclusion, UR, RP, and VA reduce epileptic seizures and also reduce the JNKp expression of the MAPK signal pathway at 3 h following KA administration, indicating that the antiepileptic effect of UR and RP is involved in initiating the JNKp MAPK signal pathway.
